# Porous Silicon-Zinc Oxide Nanocomposites Prepared by Atomic Layer Deposition for Biophotonic Applications

**DOI:** 10.3390/ma13081987

**Published:** 2020-04-24

**Authors:** Mykola Pavlenko, Valerii Myndrul, Gloria Gottardi, Emerson Coy, Mariusz Jancelewicz, Igor Iatsunskyi

**Affiliations:** 1 NanoBioMedical Centre, Adam Mickiewicz University, Wszechnicy Piastowskiej 3, 61-614 Poznan, Poland; mykpav@amu.edu.pl (M.P.); valmyn@amu.edu.pl (V.M.); coyeme@amu.edu.pl (E.C.); marjan7@amu.edu.pl (M.J.); 2 Fondazione Bruno Kessler, Center for Materials and Microsystems IRST, Via Sommarive 18, 38123 Trento, Italy; ggottard@fbk.eu

**Keywords:** porous silicon, zinc oxide, atomic layer deposition, biosensors, photoluminescence

## Abstract

In the current research, a porous silicon/zinc oxide (PSi/ZnO) nanocomposite produced by a combination of metal-assisted chemical etching (MACE) and atomic layer deposition (ALD) methods is presented. The applicability of the composite for biophotonics (optical biosensing) was investigated. To characterize the structural and optical properties of the produced PSi/ZnO nanocomposites, several studies were performed: scanning and transmission electron microscopy (SEM/TEM), X-ray diffraction (XRD), X-ray photoelectron spectroscopy (XPS), diffuse reflectance, and photoluminescence (PL). It was found that the ALD ZnO layer fully covers the PSi, and it possesses a polycrystalline wurtzite structure. The effect of the number of ALD cycles and the type of Si doping on the optical properties of nanocomposites was determined. PL measurements showed a “shoulder-shape” emission in the visible range. The mechanisms of the observed PL were discussed. It was demonstrated that the improved PL performance of the PSi/ZnO nanocomposites could be used for implementation in optical biosensor applications. Furthermore, the produced PSi/ZnO nanocomposite was tested for optical/PL biosensing towards mycotoxins (Aflatoxin B1) detection, confirming the applicability of the nanocomposites.

## 1. Introduction 

It is well known that porous silicon (PSi), due to its high surface-to-volume ratio and superior photoluminescence (PL) properties, is an attractive material for optical (bio)sensing applications [1,2]. However, the instability of PSi properties in solutions due to the degradation process needs to be solved in order to obtain a stable response and repeatable results [3]. There are numerous approaches able to reduce the degradation; among them, the deposition of metal oxide onto the PSi is considered to be the most promising one [4,5,6,7,8]. Zinc oxide (ZnO) is one of the possible candidates that could be combined with PSi because of its excellent optical properties (e.g., stable PL signal in the wide optical range), and which may be used in biophotonic applications, particularly biosensors [9,10,11]. 

Nanocomposites based on PSi/ZnO have attracted more and more attention over the years [12,13,14,15,16]. The underlying reasons behind their popularity are attributed to the synergistic effects on physical and, particularly, optical properties of the PSi/ZnO, which are derived from the combination of individual features of both semiconductors [17,18,19,20,21,22]. The improved catalytic activity, high charge carrier mobility and relatively high biocompatibility make the PSi/ZnO nanocomposite a perfect material for a reliable long-term multipurpose (bio)sensor [23,24,25,26]. Moreover, the outstanding properties of PSi/ZnO nanocomposites related to electroluminescence (EL) [27,28] and photoluminescence (PL) [29,30,31,32] can be tuned through morphology control, such as crystal size, porosity of PSi, and Si to ZnO ratio. 

The successive deposition of ZnO on PSi results in the formation of PSi/ZnO nanocomposites, whose properties are strictly affected by the specific deposition method used, such as sol-gel, chemical vapor deposition (CVD), and sputtering methods [33,34,35,36]. These methods have substantial drawbacks related to integration with CMOS technology for commercial sensors production. Moreover, these methods do not allow for the production of a conformal layer of ZnO over the whole surface of PSi or the penetration of ZnO inside the PSi matrix, features that should enhance the active area at the Si-ZnO interface. 

One of the most well developed and facile techniques for the deposition of thin metal oxide films on porous materials is atomic layer deposition (ALD) [16,37,38]. This technique allows for the fabrication of highly conformal films of ZnO and other metal oxides over the PSi surface and the production of high-quality nanocomposites in comparison to other methods [4,5,39]. Therefore, ALD is well suited for the synthesis of PSi/ZnO nanocomposites, owing to its precise control of the morphological properties of ZnO. As a consequence, it enables to tune the electronic and optical properties of produced nanocomposites.

A promising approach in the application of PSi/ZnO nanocomposites lies in photonics, particularly in biophotonics. It has been reported that PSi/ZnO nanocomposites demonstrate an intense white light emission that could be used for the development of effective light-emitting diodes [40]. The authors successively achieved a Gaussian-shape emission in the visible range through the tuning of PSi morphology in combination with the ZnO coverage. The influence of oxygen antisites, oxygen vacancies, and quantum confinement effects on the photoluminescence of PSi/ZnO has been discussed in [41], where authors also demonstrated the effect of a SiO_2_ layer grown between ZnO and PSi on the conductivity of PSi/ZnO nanocomposites. Moreover, the improvement and stabilization of photoluminescence emission have been previously shown in [42], via the passivation of n-type PSi by the deposition of a ZnO layer. 

Since ZnO emits light in the visible and UV regions, combining it with PSi leads to the formation of a nanostructure with noticeable white emission [43], which is suitable for sensors application in the visible range. Whereas the PSi/ZnO optical properties can be tuned through morphology parameters, the correlation between PL yield and the structural properties should be thoroughly investigated, especially in the context of sensor applications. D. Gallach-Pérez et al. [41] have reported on the determination of the band diagram of PSi/ZnO nanocomposites by analyzing electroluminescence (EL) spectra and I-V characteristics. It was found that PSi/ZnO broadband emission could be attributed to zinc interstitials (blue emission), oxygen vacancies (green light), and quantum confinement in Si-based quantum dots within PSi, respectively [44,45]. 

In this paper, we report on the investigation of the structural and optical properties of PSi/ZnO nanocomposites produced from p-type and n-type Si. The ZnO layer was deposited and introduced into the PSi matrix by ALD. The structural properties and chemical composition of the produced nanocomposites were characterized by scanning and transmission electron microscopy (SEM, TEM) combined with an energy dispersive X-Ray spectroscopy (EDX) analysis, an X-Ray diffraction analysis (XRD), and X-Ray photoelectron spectroscopy (XPS). The optical properties of PSi/ZnO nanocomposites were investigated by diffuse reflectance and photoluminescence (PL). The effect of the Si doping-type on the PL properties of PSi/ZnO nanocomposites was analyzed as well. A possible biophotonic application (biosensing towards mycotoxins) is also demonstrated.

## 2. Materials and Methods 

In this study, PSi/ZnO nanocomposites were produced by ALD and metal-assisted chemical etching (MACE). Samples of PSi were produced by MACE on highly doped p-type and n-type Si with (100) orientation and resistivity Ω = 0.01 ohm·cm. As it happens in a typical MACE process, silver nanoparticles were deposited on Si substrates by immersion in the metallization aqueous solution containing 0.2 M HF and 10^−3^ M AgNO_3_ for 60 s. The etching process was conducted in an aqueous solution of HF and H_2_O_2_ at ratio concentration HF/H_2_O_2_/H_2_O = 80/80/20. Then, the silver nanoparticles were removed by immersion into the HNO_3_ solution for 60 min.

A commercially available Picosun ALD reactor (Masala, Finland) was used for the deposition of ZnO. The deposition process used diethyl zinc (DEZ) and deionized water as precursors, which reacted at 200 °C and yielded a thickness of 2.1 Å per one ALD cycle (corresponding to a flat surface). The number of ALD cycles was varied as 50, 100, and 250, corresponding to a layer thickness of 10 nm, 20 nm, and 50 nm, respectively. After every cycle, the reactor chamber was purged by an intense N_2_ flow in order to remove the byproducts of chemical reactions.

The structural properties of PSi/ZnO nanocomposites were analyzed by SEM JEOL JSM 7001F and TEM JEOL ARM 200F (Tokyo, Japan). The XRD measurements were performed by X’pert3 MRD (XL) from PANalytical (Cu Kα radiation source (wavelength of 1.54 Å) and operating at 45 kV and 40 mA (Almelo, The Netherlands).

XPS spectra were recorded with a KRATOS Axis DLD Ultra instrument (Kratos–Manchester, UK) equipped with a hemispherical analyzer and a monochromatic Al Kα (1486.6 eV) X-ray source. The broad spectra (survey) were acquired at 160 eV pass energy, while a higher resolution was used for the acquisition of the core lines, which was performed by setting the pass energy at 20 eV. This allowed to reach an energy resolution of ~0.4 eV. The analyses were acquired at 90° of samples tilted with respect to the analyzer. The spectra were fitted using a freely available software (R-XPS) based on an R-platform (G.Speranza/RxpsG software, RxpsG-2.1 version) [46].

The diffuse reflectance measurements of PSi/ZnO nanocomposites were performed by Ocean Optics QE PRO fiber optic spectrometer (Ostfildern, Germany) combined with an integrating sphere and a Xe light source. Acquisition of PL data was performed at room temperature using a He-Cd laser from Kimmon Koha (Tokyo, Japan) with a wavelength of 325 nm and an output of 5 mW as an excitation source in the range of 400–1000 nm.

## 3. Results and Discussion

### 3.1. Structural Properties of PSi/ZnO Nanocomposites 

**Figure 1:**
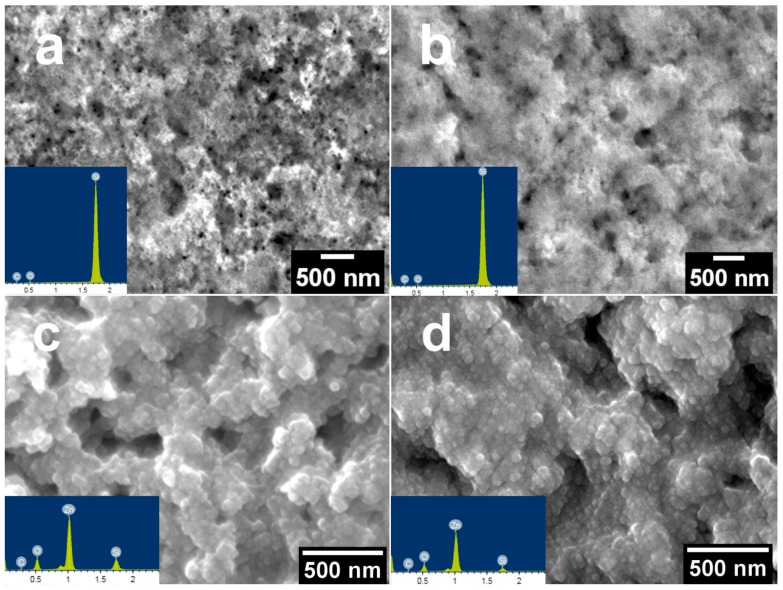
SEM images of porous silicon (PSi) surface before and after 100 cycles of zinc oxide ALD: (**a**) n-type PSi; (**b**) p-type PSi; (**c**,**d**) n-and p-types PSi after zinc oxide (ZnO) deposition, respectively (insets: an EDX analysis).

SEM and EDX were performed in order to study the morphology and elemental composition of the produced nanocomposites. The SEM images of PSi and PSi/ZnO nanocomposites derived from p- and n-types Si are represented in Figure 1. Both types of produced PSi samples (Figure 1a,b) show a mesoporous structure (the average size of pores ranged from 10 to 50 nm) with a uniform distribution of the pores. The slightly different roughness of PSi is clearly visible, depending on the type of Si used as a substrate. This might be explained by the different hole concentration during the MACE process, which strongly affects the etching process and as a consequence, defines the surface roughness [47].

After the ALD process, we can observe that the ZnO layer fully covers the PSi (Figure 1c,d). One may also note that the ZnO layer consists of uniformly distributed grains. In addition, the analyses demonstrate that ZnO infiltrates into the PSi matrix. EDX confirms the presence of silicon, oxygen, and zinc atoms after ALD (see Figure 1 insets). EDX mapping indicates the homogeneous distribution of Zn over the whole PSi surface (not shown here).

**Figure 2:**
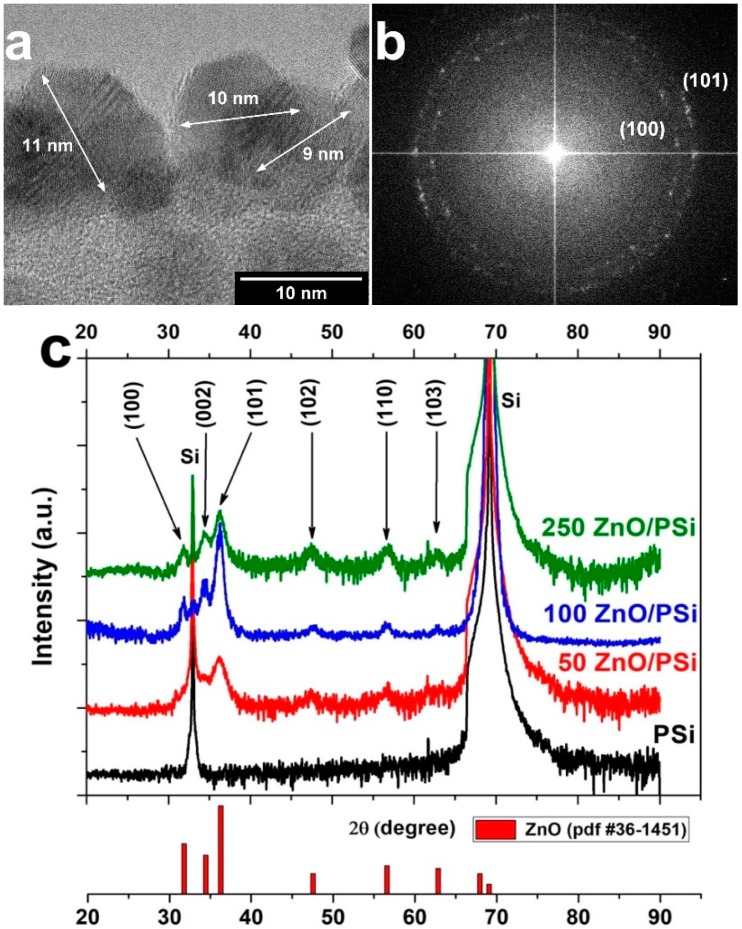
(**a**) TEM imaging of ZnO nanocrystallites on PSi obtained after 100 ALD cycles; (**b**) FFT of the PSi/ZnO TEM image; (**c**) GIXRD spectra of PSi/ZnO nanocomposites with 250, 100, and 50 ALD cycles of ZnO in comparison to GIXRD spectrum of PSi. Standard diffraction peaks of wurtzite (pdf card #36-1451) are presented for reference.

Figure 2a shows a TEM image of PSi after 100 ALD cycles of ZnO, which correspond to 20 nm of layer thickness. In the TEM image, the presence of ZnO nanocrystallites can be observed. The average size of ZnO nanocrystallites is about 9–11 nm. This result is similar to the value obtained previously for PSi/metal oxide nanocomposites [16,48], once again proving the reproducibility of the method. The Fast Fourier Transform analysis (FFT) shows the highly polycrystalline nature of the deposited ZnO layer, where the wurtzite phase with prevailing (101) and (100) orientations can be indexed.

To determine the structural properties of the PSi/ZnO nanocomposites, XRD measurements were performed. Figure 2c shows the XRD spectra collected from PSi/ZnO with different numbers of ALD cycles: 50, 100, and 250. A clear wurtzite phase pattern can be identified for all samples, represented by (100), (002), (101), (102), (110), and (103) peaks. Some of these peaks are barely detectable for 50 ALD cycles. However, upon increasing the number of ALD cycles, the intensity of wurtzite peaks increases, and the full half maximum (FWHM) is reduced. This confirms the improvement of the ZnO layer crystallinity. Average crystalline sizes were calculated using the Scherrer equation [16,48] and estimated as 6.4 ± 1.5 nm, 7.8 ± 1.2 nm, and 8 ± 1.5 nm for samples deposited with 50, 100, and 250 ALD cycles, respectively. The obtained values corroborate with the experimental data provided by the TEM micrographs.

### 3.2. XPS Studies 

**Figure 3:**
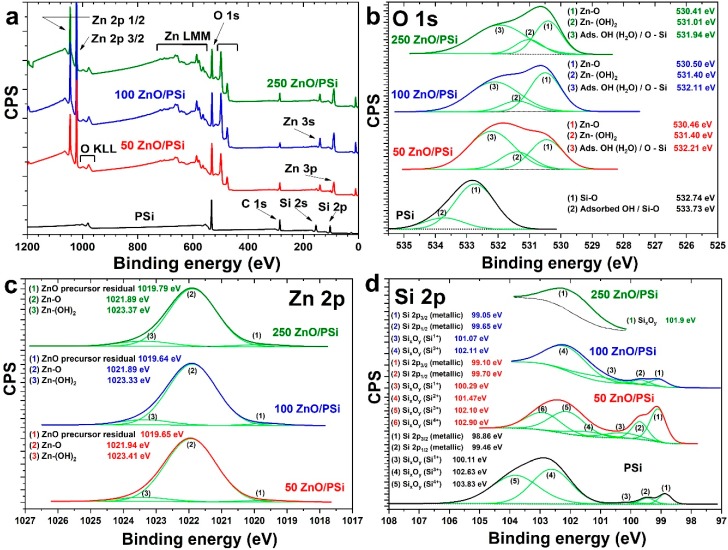
XPS survey and core-level spectra of PSi/ZnO with 50, 100, and 250 ALD cycles: (**a**) total survey spectra; (**b**–**d**) O 1s, Zn 2p, Si 2p energy regions, respectively. Corresponding binding energy values obtained by the deconvolution of the detected peaks are shown in the insets.

The chemical composition of the produced PSi/ZnO nanocomposites was analyzed by XPS. The survey spectra of PSi and PSi/ZnO samples deposited with different number of ALD cycles are presented in Figure 3a. The surface composition of PSi is primarily dominated by the signals of silicon and oxygen elements. The PSi sample surface displays an abundant contamination of carbon, which originates from the MACE process. All PSi/ZnO samples’ wide spectra are characterized, on the other hand, by the Zn 3p, Zn 3s, O 1s, Zn 2p_3/2,_ and Zn 2p_1/2_ peaks and the Zn LMM and O KLL Auger peaks. The pattern of survey spectra is generally common for the measured PSi/ZnO samples, but the stoichiometry of the ZnO layer is expected to change with the number of ALD cycles. By considering the XPS high-resolution core lines with their respective experimental sensitivity factors (RSF), the ZnO layer stoichiometry (ratio O/Zn) was determined (Table 1). There is a clear increment of the ZnO stoichiometry with the increase of ALD cycles (the layer thickness). The lower values of the O/Zn for the 50 PSi/ZnO sample could be explained by the high concentration of Zn defects in the deposited layer.

Figure 3b compares the O 1s energy region of the different samples. The O 1s core level peak of as-prepared PSi was deconvoluted into two distinct components at 532.74 eV and 533.73 eV. The first component corresponds to the oxidized PSi surface, while the high-energy component is attributed to absorbed environmental moisture [49]. On the other hand, deconvolution of O 1s peak on samples covered by ZnO shows three components, which are related to Zn-O (530.45 eV) bounds, Zn-(OH)_2_ (531.40 eV) species, and OH groups (532.1 eV) adsorbed during the ALD process [50]. The intensities and positions of the revealed components of O 1s core line spectra change slightly with the number of ALD cycles. The component responsible for adsorbed water and Si-O bonds is predominant in the 50 ZnO/PSi samples, and noticeably declined in the other samples. This feature can be explained by an increase of ZnO moiety in samples with 100 and 250 cycles and resulted in an increment in Zn-O components.

The detailed spectra of Zn 2p core levels are presented in Figure 3c. The Zn 2p_3/2_ peak was fitted with two main components located at 1021.89 eV and 1023.37 eV (corresponding to Zn-O and Zn-(OH)_2_) and an extremely weak third component at 1019.79 eV, which may be attributed to some residuals of unreacted Zn precursor [50]. Attribution of the component at 1023.37 eV to hydroxide species is corroborated by the O 1s peak at 531.4 eV. The presence of metal oxide-OH species on the surface has been previously observed for ZnO as well as for TiO_2_, Al_2_O_3,_ and RuO_2_ produced by ALD [5,39,49,50]. One may conclude that this is a feature of produced ALD metal oxides. 

No signal from Si is clearly visible from the survey of PSi/ZnO nanocomposites. Its presence was checked by acquiring a detailed spectrum in the region of Si 2p core line, in order to verify if a small signal coming from the surface oxide was still visible, even after the deposition of 250 cycles of ZnO. In the Si 2p core line (Figure 3d), it is clearly visible in the metallic component with the spin-orbit doublet, which can be easily resolved into its 2p_1/2_ and 2p_3/2_ contributions. The silicon oxide layer generates a broad peak at higher binding energies, which can be resolved by overlapping at least 3 components, due to the different possible oxidation states of Si (Si^1+^, Si^2+^, Si^3+^ or Si^4+^). A more visible signal coming from the silicon substrate can be detected by decreasing the number of ALD cycles, and both the metallic doublet and the oxide components can be distinguished.

### 3.3. Optical and Biosensing Properties

In order to elucidate the optical properties of the produced PSi/ZnO nanocomposites, the diffuse reflectance and the photoluminescence were measured in the ranges of 300–750 nm and 400–1000 nm, respectively.

**Figure 4:**
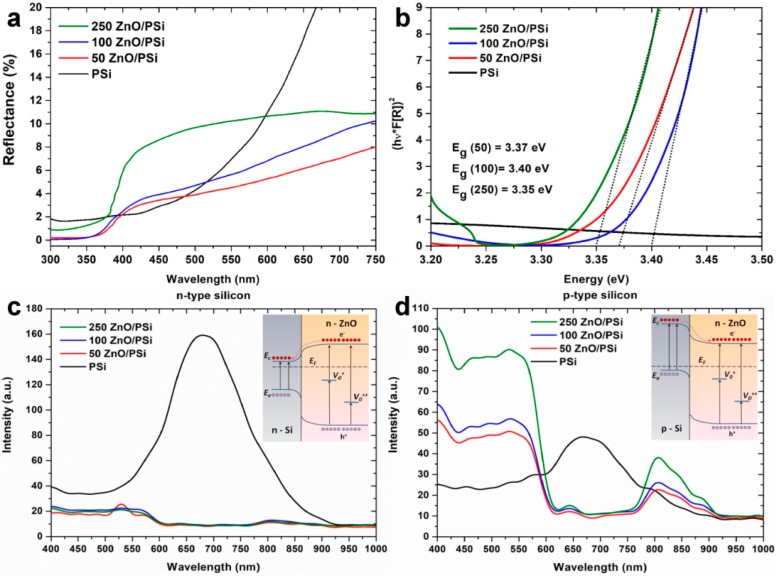
Optical properties of the fabricated PSi/ZnO nanocomposites: (**a**) diffuse reflectance spectra of PSi/ZnO nanocomposites; (**b**) absorption edges and corresponding energy band gap values; photoluminescence spectra for (**c**) n-PSi/ZnO and (**d**) p-PSi/ZnO nanocomposites. The energy band diagrams of corresponding excitation mechanisms are depicted in the insets.

Diffuse reflectance spectra for as-prepared PSi and PSi/ZnO with 50, 100, and 250 ALD cycles are shown in Figure 4a. All the samples show a significantly low reflectance in the UV region, and a noticeable increase in the visible region up to 10% for 250 cycles of ZnO. Recalculation of the obtained data into a representation of a Tauc plot was performed according to the Kubelka-Munk theory via F(R) function: $F\left(R\right)=\frac{{\left(1-R\right)}^{2}}{2R}=\frac{k}{S}$ [5], where *R* is the absolute reflectance of the PSi/ZnO sample, *k* is the absorption coefficient, and *s* is the scattering coefficient (Figure 4b). The calculated band gap (E_g_) values are 3.37 eV, 3.40 eV, and 3.35 eV for samples with 50, 100, and 250 ALD cycles, respectively. Such values are close to the bulk ZnO E_g_ value, thus proving the good crystallinity of the produced ALD layers.

PL measurements were conducted to investigate the recombination processes in the Si-ZnO interface. PL spectra were measured for PSi/ZnO nanocomposites produced from n- and p-type of Si (Figure 4c,d). The as-prepared PSi demonstrates a strong red PL emission at approximately 670 nm (1.85 eV) for both types of Si. It is well known that the visible emission of PSi is attributed to the quantum confinement effect of Si nanocrystallites, which are formed during etching processes [51,52]. After the ZnO deposition, the PL spectrum changes depending on the type of initial Si. Figure 4c demonstrates the PL quenching for n-type PSi/ZnO nanocomposites. However, one may observe significant PL changes for p-type PSi/ZnO nanocomposites (Figure 4d). The p-type PSi/ZnO nanocomposites demonstrate an intensive white PL emission with a strong “shoulder-shape” contribution in the 400–600 nm region, and a weak Infra-red (IR) emission in the 800–900 nm region. The “shoulder-shape” PL in the visible range probably originates from the complex mechanism of the recombination emission through defect levels of ZnO, which may be attributed to radiative recombination at single and double-charged oxygen vacancy (Vo^+^, Vo^++^) sites [35,53]. However, the full interpretation and explanation of observed PL will be the aim of future research. 

One could propose a simpler explanation of the observed experimental results based on the analysis of energy band diagrams for the Si-ZnO interface (insets in Figure 4c,d). It is well known that oxygen vacancies (depicted as Vo^+^, Vo^++^ in the energy band diagrams) are the most widely accepted mechanisms of the visible emission in ZnO. Let us assume that all laser radiation is adsorbed in the top ZnO layer. In the case of n-type PSi/ZnO, according to the energy band diagrams, it can be seen that the electrons and holes photogenerated in the ZnO layer should be drifted to the Si. These photogenerated charge carriers are recombined through non-radiative surface defects, probably associated with SiO_x_, the presence of which was confirmed by XPS. In the case of p-type PSi/ZnO, the interface of p- and n-type semiconductors induces the formation of a potential barrier for photogenerated electrons in ZnO (see inset Figure 4d). Thus, photogenerated charge carriers tend to recombine through defect levels of ZnO. An increased intensity of PL around the 400 nm edge probably originates from the exciton recombination in ZnO [16]. The presence of red/IR PL may be explained by additional defect states on the surface of n-PSi/ZnO nanocomposite [34,54]. 

The PL analysis demonstrates that p-type PSi/ZnO nanocomposites are more favorable for the development of novel biophotonic (e.g., optical biosensors) devices. This nanocomposite was tested for optical/PL biosensing applications towards mycotoxins (Aflatoxin B1) detection. We analyzed the evaluation of the PL spectrum, depending on the concentration of AFB1 probed on the surface. The formation of the bioselective layer for AFB1 was performed according to the protocol based on previous works [55,56]. Briefly, the PSi/ZnO was treated with 4% APTES in Ethanol with the following carboxyl groups activation in a solution of 2% glutaraldehyde in distilled water. In the next step, 50 μg/mL of Anti-AFB1 (in PBS) was added to achieve a selective layer to AFB1. Before the experiment, the samples were washed with PBS and gently dried using an N_2_ flow. Then, the probe of the PBS/AFB1 solution (1, 10, and 100 ng/mL) was dropped onto the surface of the PSi/ZnO nanocomposite and washed with PBS after 20 min.

**Figure 5:**
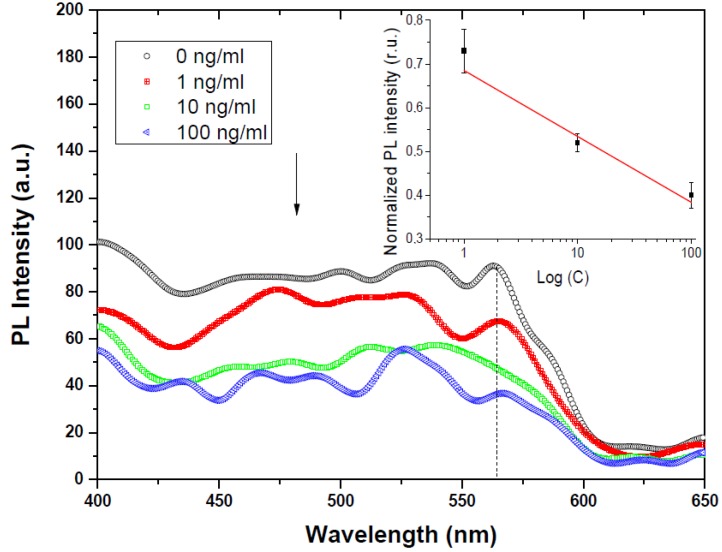
PL response of p-type PSi/ZnO toward different AFB1 concentrations. The inset graph indicates the linearity of PL response to three different concentrations of AFB1 in a half-logarithmic scale with an error bar.

Figure 5 represents the PL response of PSi/ZnO nanocomposite in comparison to different concentrations of AFB1. The PL-based detection of AFB1 on the PSi/ZnO nanostructure shows sequential PL (λ = 565 nm) quenching from the lower to the highest AFB1 concentration, which makes the PSi/ZnO nanocomposite a suitable material for further usage in real-time (bio)molecule detection. Furthermore, the inset in Figure 5 indicates a good linearity of sensor response, which is the key parameter for the development of effective biosensors [57]. Thus, based on the evidence mentioned above, PSi/ZnO could be used as a significant potential optical biosensing platform for different types of biomolecules.

## 4. Conclusions

In summary, PSi/ZnO nanocomposites were fabricated using MACE and ALD techniques. Their structural properties and chemical compositions were determined. The approximate size of ZnO nanograins was estimated using XRD and TEM analysis. The effect of the number of ALD cycles and the type of Si used as substrate on the optical properties of nanocomposites were studied. Optical properties (reflectance and band gap energy) of PSi/ZnO nanocomposites were tailored by their structural parameters. It was demonstrated that an effective white “shoulder-shape” may be used for implantation in optical biosensor applications toward mycotoxins (Aflatoxin B1) detection, as a model molecule.

## Figures and Tables

**Table 1:** ZnO layer stoichiometry.

Sample	Zn (in Zn-O), at. %	O (in Zn-O), at %	O/Zn
250 PSi/ZnO	17.85	18.21	1.02
100 PSi/ZnO	20.54	20.38	0.99
50 PSi/ZnO	15.48	14.00	0.90

## References

[1] Myndrul V., Iatsunskyi I. (2019). Nanosilicon-Based Composites for (Bio)sensing Applications: Current Status, Advantages, and Perspectives. Materials.

[2] Iatsunskyi I., Smyntyna V., Pavlenko M., Kanevska O., Kirik Y., Myndrul V. Ammonia detection using optical reflectance from porous silicon formed by metal-assisted chemical etching. Proceedings of the Optics and Photonics for Counterterrorism, Crime Fighting and Defence IX; and Optical Materials and Biomaterials in Security and Defence Systems Technology X.

[3] Tischler M.A., Collins R.T., Stathis J.H., Tsang J.C. (1992). Luminescence degradation in porous silicon. Appl. Phys. Lett..

[4] Iatsunskyi I., Jancelewicz M., Nowaczyk G., Kempiński M., Peplińska B., Jarek M., Załęski K., Jurga S., Smyntyna V. (2015). Atomic layer deposition TiO_2_ coated porous silicon surface: Structural characterization and morphological features. Thin Solid Films.

[5] Brytavskyi I., Hušeková K., Myndrul V., Pavlenko M., Coy E., Zaleski K., Gregušová D., Yate L., Smyntyna V., Iatsunskyi I. (2019). Effect of porous silicon substrate on structural, mechanical and optical properties of MOCVD and ALD ruthenium oxide nanolayers. Appl. Surf. Sci..

[6] Silina Y.E., Koch M., Herbeck-Engel P., Iatsunskyi I. (2019). Exploring the potential of high resolution inductively coupled plasma mass spectrometry towards non-destructive control and validation of electroless gold nanoparticles onto silicon nanowires hybrids. Anal. Methods.

[7] Pavlenko M., Siuzdak K., Coy E., Jancelewicz M., Jurga S., Iatsunskyi I. (2017). Silicon/TiO_2_ core-shell nanopillar photoanodes for enhanced photoelectrochemical water oxidation. Int. J. Hydrogen Energy.

[8] Pavlenko M., Coy E.L., Jancelewicz M., Załȩski K., Smyntyna V., Jurga S., Iatsunskyi I., Zaleski K., Smyntyna V., Jurga S. (2016). Enhancement of optical and mechanical properties of Si nanopillars by ALD TiO_2_ coating. RSC Adv..

[9] Viter R., Savchuk M., Iatsunskyi I., Pietralik Z., Starodub N., Shpyrka N., Ramanaviciene A., Ramanavicius A. (2018). Analytical, thermodynamical and kinetic characteristics of photoluminescence immunosensor for the determination of Ochratoxin A. Biosens. Bioelectron..

[10] Tamashevski A., Harmaza Y., Viter R., Jevdokimovs D., Poplausks R., Slobozhanina E., Mikoliunaite L., Erts D., Ramanaviciene A., Ramanavicius A. (2019). Zinc oxide nanorod based immunosensing platform for the determination of human leukemic cells. Talanta.

[11] Viter R., Savchuk M., Starodub N., Balevicius Z., Tumenas S., Ramanaviciene A., Jevdokimovs D., Erts D., Iatsunskyi I., Ramanavicius A. (2019). Photoluminescence immunosensor based on bovine leukemia virus proteins immobilized on the ZnO nanorods. Sens. Actuators B Chem..

[12] Rusli N.I., Tanikawa M., Mahmood M.R., Yasui K., Hashim A.M. (2012). Growth of high-density zinc oxide nanorods on porous silicon by thermal evaporation. Materials.

[13] Hsu H.-C., Cheng C.-S., Chang C.-C., Yang S., Chang C.-S., Hsieh W.-F. (2005). Orientation-enhanced growth and optical properties of ZnO nanowires grown on porous silicon substrates. Nanotechnology.

[14] Kanungo J., Saha H., Basu S. (2010). Pd sensitized porous silicon hydrogen sensor—Influence of ZnO thin film. Sens. Actuators B Chem..

[15] Yan D., Hu M., Li S., Liang J., Wu Y., Ma S. (2014). Electrochemical deposition of ZnO nanostructures onto porous silicon and their enhanced gas sensing to NO2 at room temperature. Electrochim. Acta.

[16] Graniel O., Fedorenko V., Viter R., Iatsunskyi I., Nowaczyk G., Weber M., Załęski K., Jurga S., Smyntyna V., Miele P. (2018). Optical properties of ZnO deposited by atomic layer deposition (ALD) on Si nanowires. Mater. Sci. Eng. B.

[17] Wang Z., Yu R., Wang X., Wu W., Wang Z.L. (2016). Ultrafast Response p-Si/n-ZnO Heterojunction Ultraviolet Detector Based on Pyro-Phototronic Effect. Adv. Mater..

[18] Huang C.-Y., Yang Y.-J., Chen J.-Y., Wang C.-H., Chen Y.-F., Hong L.-S., Liu C.-S., Wu C.-Y. (2010). p-Si nanowires/SiO_2_/n-ZnO heterojunction photodiodes. Appl. Phys. Lett..

[19] Guo Z., Zhao D., Liu Y., Shen D., Zhang J., Li B. (2008). Visible and ultraviolet light alternative photodetector based on ZnO nanowire/n-Si heterojunction. Appl. Phys. Lett..

[20] Choi J.-H., Das S.N., Moon K.-J., Kar J.P., Myoung J.-M. (2010). Fabrication and characterization of p-Si nanowires/ZnO film heterojunction diode. Solid State Electron..

[21] Zhang H., Jia Z. (2017). Application of porous silicon microcavity to enhance photoluminescence of ZnO/PS nanocomposites in UV light emission. Optik (Stuttg).

[22] Shabnam, Kant C.R., Arun P. (2012). White-light emission from annealed ZnO:Si nanocomposite thin films. J. Lumin..

[23] Shanmugam N.R., Muthukumar S., Prasad S. (2017). A review on ZnO-based electrical biosensors for cardiac biomarker detection. Future Sci. OA.

[24] Tripathy N., Kim D.-H. (2018). Metal oxide modified ZnO nanomaterials for biosensor applications. Nano Converg..

[25] Napi M.L.M., Sultan S.M., Ismail R., How K.W., Ahmad M.K. (2019). Electrochemical-Based Biosensors on Different Zinc Oxide Nanostructures: A Review. Materials.

[26] Sampath S., Shestakova M., Maydannik P., Ivanova T., Homola T., Bryukvin A., Sillanpää M., Nagumothu R., Alagan V. (2016). Photoelectrocatalytic activity of ZnO coated nano-porous silicon by atomic layer deposition. RSC Adv..

[27] Sun H., Zhang Q.-F., Wu J.-L. (2006). Electroluminescence from ZnO nanorods with an n-ZnO/p-Si heterojunction structure. Nanotechnology.

[28] Ye J.D., Gu S.L., Zhu S.M., Liu W., Liu S.M., Zhang R., Shi Y., Zheng Y.D. (2006). Electroluminescent and transport mechanisms of n-ZnO/p-Si heterojunctions. Appl. Phys. Lett..

[29] Palanivelu R., Rubankumar A. (2013). Synthesis and Spectroscopic Characterization of Hydroxyapatite by Sol-Gel Method. Int. J. ChemTech Res..

[30] Singh R.G., Singh F., Agarwal V., Mehra R.M. (2007). Photoluminescence studies of ZnO/porous silicon nanocomposites. J. Phys. D Appl. Phys..

[31] Ambrosio R., Galindo F., Morales–Morales F., Moreno M., Torres A., Vásquez-A M.A., Pérez García S.A., Morales–Sánchez A. (2019). Effect of the thermal annealing on the structural, morphological and photoluminescent properties of ZnO/Si multilayers. Opt. Mater..

[32] Olenych I.B., Monastyrskii L.S., Luchechko A.P. (2017). Photoluminescence of Porous Silicon–Zinc Oxide Hybrid structures. J. Appl. Spectrosc..

[33] Sun L., He H., Liu C., Lu Y., Ye Z. (2011). Controllable growth and optical properties of ZnO nanostructures on Si nanowire arrays. CrystEngComm.

[34] Dalvand R., Mahmud S., Alimanesh M., Vakili A.H. (2017). Optical and structural properties of well-aligned ZnO nanoneedle arrays grown on porous silicon substrates by electric field-assisted aqueous solution method. Ceram. Int..

[35] Singh R.G., Singh F., Kanjilal D., Agarwal V., Mehra R.M. (2009). White light emission from chemically synthesized ZnO–porous silicon nanocomposite. J. Phys. D Appl. Phys..

[36] Graniel O., Iatsunskyi I., Coy E., Humbert C., Barbillon G., Michel T., Maurin D., Balme S., Miele P., Bechelany M. (2019). Au-covered hollow urchin-like ZnO nanostructures for surface-enhanced Raman scattering sensing. J. Mater. Chem. C.

[37] Iatsunskyi I., Baitimirova M., Coy E., Yate L., Viter R., Ramanavicius A., Jurga S., Bechelany M., Erts D. (2018). Influence of ZnO/graphene nanolaminate periodicity on their structural and mechanical properties. J. Mater. Sci. Technol..

[38] Titov V.V., Lisachenko A.A., Akopyan I.K., Labzowskaya M.E., Novikov B.V. (2018). On the nature of the effect of adsorbed oxygen on the excitonic photoluminescence of ZnO. J. Lumin..

[39] Iatsunskyi I., Kempiński M., Jancelewicz M., Załęski K., Jurga S., Smyntyna V. (2015). Structural and XPS characterization of ALD Al_2_O_3_ coated porous silicon. Vacuum.

[40] Dellis S., Pliatsikas N., Kalfagiannis N., Lidor-Shalev O., Papaderakis A., Vourlias G., Sotiropoulos S., Koutsogeorgis D.C., Mastai Y., Patsalas P. (2018). Broadband luminescence in defect-engineered electrochemically produced porous Si/ZnO nanostructures. Sci. Rep..

[41] Gallach-Pérez D., Muñoz-Noval A., García-Pelayo L., Manso-Silván M., Torres-Costa V. (2017). Tunnel conduction regimes, white-light emission and band diagram of porous silicon–zinc oxide nanocomposites. J. Lumin..

[42] Prabakaran R., Peres M., Monteiro T., Fortunato E., Martins R., Ferreira I. (2008). The effects of ZnO coating on the photoluminescence properties of porous silicon for the advanced optoelectronic devices. J. Non-Cryst. Solids.

[43] Kayahan E. (2010). White light luminescence from annealed thin ZnO deposited porous silicon. J. Lumin..

[44] Algün G., Akçay N. (2019). The role of etching current density and porous structure on enhanced photovoltaic performance of ZnO/PS heterojunction solar cells. Appl. Phys. A.

[45] Aydemir G., Utlu G., Çetinel A. (2019). Growth and characterization of ZnO nanostructures on porous silicon substrates: Effect of solution temperature. Chem. Phys. Lett..

[46] Speranza G., Canteri R. (2019). RxpsG a new open project for Photoelectron and Electron Spectroscopy data processing. SoftwareX.

[47] Han H., Huang Z., Lee W. (2014). Metal-assisted chemical etching of silicon and nanotechnology applications. Nano Today.

[48] Iatsunskyi I., Pavlenko M., Viter R., Jancelewicz M., Nowaczyk G., Baleviciute I., Załęski K., Jurga S., Ramanavicius A., Smyntyna V. (2015). Tailoring the Structural, Optical, and Photoluminescence Properties of Porous Silicon/TiO_2_ Nanostructures. J. Phys. Chem. C.

[49] Iatsunskyi I., Kempiński M., Nowaczyk G., Jancelewicz M., Pavlenko M., Załęski K., Jurga S. (2015). Structural and XPS studies of PSi/TiO_2_ nanocomposites prepared by ALD and Ag-assisted chemical etching. Appl. Surf. Sci..

[50] Iatsunskyi I., Vasylenko A., Viter R., Kempiński M., Nowaczyk G., Jurga S., Bechelany M. (2017). Tailoring of the electronic properties of ZnO-polyacrylonitrile nanofibers: Experiment and theory. Appl. Surf. Sci..

[51] Bisi O., Ossicini S., Pavesi L. (2000). Porous silicon: A quantum sponge structure for silicon based optoelectronics. Surf. Sci. Rep..

[52] Iatsunskyi I., Nowaczyk G., Jurga S., Fedorenko V., Pavlenko M., Smyntyna V. (2015). One and two-phonon Raman scattering from nanostructured silicon. Optik.

[53] Marin O., Grinblat G., María A., Tirado M., Koropecki R.R., Comedi D. (2015). Superlattices and Microstructures On the origin of white photoluminescence from ZnO nanocones/porous silicon heterostructures at room temperature. Superlattices Microstruct..

[54] Ma Q.L., Zhai B.G., Huang Y.M. (2012). Sol-gel derived ZnO/porous silicon composites for tunable photoluminescence. J. Sol-Gel Sci. Technol..

[55] Iatsunskyi I., Myndrul V., Smyntyna V., Viter R., Melnyk Y., Pavlova K. (2017). Porous silicon photoluminescence biosensor for rapid and sensitive detection of toxins. Org. Sens. Bioelectron. X.

[56] Myndrul V., Viter R., Savchuk M., Koval M., Starodub N., Silamiķelis V., Smyntyna V., Ramanavicius A., Iatsunskyi I. (2017). Gold coated porous silicon nanocomposite as a substrate for photoluminescence-based immunosensor suitable for the determination of Aflatoxin B1. Talanta.

[57] Viter R., Iatsunskyi I., Zenkina O.V. (2019). Metal Oxide Nanostructures in Sensing. Nanomaterials Design for Sensing Applications.

